# Genome sequencing in a cohort of 32 fetuses with genetic skeletal disorders

**DOI:** 10.1038/s41431-025-01886-x

**Published:** 2025-06-11

**Authors:** Hillevi Lindelöf, Anna Hammarsjö, Ulrika Voss, Serena Gaetana Piticchio, Peter Conner, Nikos Papadogiannakis, Dominyka Batkovskyte, Laura Orellana, Malin Kvarnung, Helena Malmgren, Kristina Lagerstedt Robinson, Ann Nordgren, Anna Lindstrand, Gen Nishimura, Giedre Grigelioniene

**Affiliations:** 1 Department of Molecular Medicine and Surgery, Karolinska Institutet, Stockholm, Sweden; 2 Department of Clinical Genetics and Genomics, Karolinska University Hospital, Stockholm, Sweden; 3 Department of Pediatric Radiology, Karolinska University Hospital, Stockholm, Sweden; 4 Protein Dynamics and Mutation lab, Department of Oncology-Pathology, Karolinska Institutet, Stockholm, Sweden; 5 Center for Fetal Medicine, Karolinska University Hospital, Stockholm, Sweden; 6 Department of Laboratory Medicine, Division of Pathology, Karolinska Institutet, Stockholm, Sweden; 7 Department of Radiology, Musashino-Yowakai Hospital, Tokyo, Japan

**Keywords:** Genetics research, Disease genetics, Genetic testing

## Abstract

Approximately 200 genetic skeletal disorders can present prenatally, detectable through ultrasound abnormalities during pregnancy. Severe forms are typically identified during the first or second trimester, whereas milder phenotypes are recognized later, in the third trimester. Diagnosing skeletal dysplasia prenatally is challenging due to the large number of disorders and the overlapping clinical findings that can be detected by ultrasound. This study, conducted at Karolinska University Hospital between 2015 and 2022, examines the genetic and radiographic findings in 32 fetuses (14 female and 18 male, from unrelated families) with skeletal abnormalities detected on prenatal ultrasound and confirmed by radiographs at birth or after pregnancy termination. Fetal DNA samples from all 32 fetuses underwent singleton genome sequencing using an in silico skeletal dysplasia gene panel. As a second step, for six fetuses with molecularly unsolved diagnoses, trio genome sequencing analysis involving the fetus and both parents was performed. The diagnostic yield of genome sequencing was 72%, with pathogenic or likely pathogenic variants identified in 23 of the 32 fetuses. Additionally, four variants of uncertain significance, strongly suspected to be causative based on clinical and radiographic features, as well as structural protein analyses, were identified in four fetuses with autosomal recessive conditions. The diagnoses of five fetuses remain molecularly unsolved. In conclusion, by combining detailed phenotypic data with singleton genome sequencing we were able to reach a genetic diagnosis in 72% of 32 fetal genetic skeletal disorder cases investigated at the Karolinska University Hospital.

## Introduction

Genetic skeletal disorders are a large group of rare conditions that affect the development and growth of the skeleton. With 771 identified clinical entities, each disorder possesses unique characteristics, inheritance patterns, and prognosis [1]. Among these, approximately 200 disorders are identifiable prenatally, presenting challenges for early detection and management. These disorders vary widely in their phenotypic presentation, ranging from mild anomalies often recognized in the third trimester, to severe, life-limiting malformations that are diagnosed already in the first or second trimester. Traditional prenatal screening methods, such as ultrasound scans, are crucial for the initial detection of skeletal abnormalities. However, the information obtained from fetal ultrasound often does not provide enough detail and there is a large overlap in the clinical findings between different conditions. Although fetal CT offers more detailed imaging and has been effectively utilized as an additional method in some countries [2], its implementation in Sweden remains limited due to concerns regarding radiation exposure. The widespread availability of massive parallel sequencing, including its compatibility with low-input sequencing technologies, has made prenatal genetic testing on DNA from amniotic fluid or chorionic villus samples a valuable diagnostic complement. By examining 32 fetuses with prenatally detected skeletal abnormalities confirmed by postnatal radiographs, we aimed to determine the diagnostic yield and identify previously unreported genetic variants contributing to these disorders, to improve prenatal diagnostic accuracy and enhance genetic counseling.

## Materials and methods

### Study subjects

The cohort includes 32 unrelated fetuses (14 female and 18 male), referred for diagnostic genetic testing at the Department of Clinical Genetics and Genomics, Karolinska University Hospital, Sweden, between 2015 and 2022. Fetuses were included if they underwent genome sequencing for a suspected skeletal dysplasia, and radiographs taken postmortem or at birth showed findings consistent with a genetic skeletal disorder. Fetal cases with clinical requests for specific monogenic disorders like thanatophoric dysplasia (TD1, TD2), achondroplasia or campomelic dysplasia, are typically screened for the requested genes using Sanger sequencing or targeted exome sequencing (ES), and were excluded from this cohort (*n* = 10). For fetus 18 with achondroplasia, a broader skeletal dysplasia panel was used instead of targeted testing due to inconclusive prenatal ultrasound findings. The radiographs were evaluated by two pediatric radiologists with expertise in diagnosing skeletal dysplasias. Fetal malformations were detected during prenatal obstetrical ultrasound scans performed to monitor fetal development, growth, and detect abnormalities. They were conducted at various stages of gestation, typically as a routine second trimester scan between 18 and 20 weeks, or as part of the first trimester scan, which is performed between 11 and 13 weeks +6 days of pregnancy. All but one fetus had skeletal abnormalities detected during prenatal ultrasound scans. The exception was fetus number 24, with focal dermal hypoplasia, *PORCN*-related (OMIM#305600), which resulted in intrauterine fetal death at 29 weeks of gestation (Table 1). Of the fetuses, 29 ended with either termination of pregnancy (TOP) or pregnancy loss, and three pregnancies resulted in live births (numbers 28, 18 and 21). Autopsies were performed in 29 of the fetuses; one was excluded at the family’s request (number 22). DNA was isolated from amniocentesis (6/32) or chorionic villus biopsy (CVB, 5/32) during pregnancy, or from fetal tissue postmortem (19/32 lung, 1/32 skin and 1/32 unspecified fetal tissue). At the time of the initial genetic testing, the gestational ages ranged from 12 to 29 weeks, maternal ages 21–44 years (*n* = 32, with an average of 32 years), and paternal ages 25–51 years (*n* = 26, with an average of 34 years). Paternal ages were unknown in six cases. Peripheral venous blood samples were collected from 50 of 64 parents for segregation or trio analysis. Supplementary Fig. S1 summarizes the flowchart of the study.

**Table 1:** Overview of molecularly solved fetuses in the cohort.

Nr	Sex	GA	Tissue	Name of disorder (Nosology 2023 revision)	Group nr^a^	Gene	Refseq	Zyg	Variant type	cDNA	Protein	ACMG^b^	Variant ref	Inh	Heredity	Radiographs^c^
1	F	18+	AF	Hypophosphatasia, *ALPL*-related, recessive (biallelic) forms	27-0010	*ALPL*	NM_000478.6	Het comp	SNV	c.[370A>G] c.[459G>A]	p.(Asn124Asp) p.(Trp153*)	LP P	VarID:1721046 VarID:1387764	AR	Pa Ma	No
2	M	13+	Lung	Osteogenesis imperfecta, severe perinatal form (Sillence type 2) *COL1A1*-related	26-0030	*COL1A1*	NM_000088.4	Het	SNV	c.2696G>A	p.(Gly899Asp)	LP	VarID:988360	AD	De novo	No
3	F	21 + 5	Lung	Osteogenesis imperfecta, progressively deforming (Sillence type 3), *COL1A2*-related	26-0090	*COL1A2*	NM_000089.4	Het	SNV	c.3287G>C	p.(Gly1096Ala)	P	VarID:988368	AD	De novo	No
4	F	18+	Lung	Osteogenesis imperfecta, severe perinatal form (Sillence type 2) *COL1A1*-related	26-0030	*COL1A2*	NM_000089.4	Het	SNV	c.2187+1G>C	p.?	P	VarID:3363158	AD	De novo	No
5	F	19+	Lung	Osteogenesis imperfecta, severe perinatal form (Sillence type 2) *COL1A1*-related	26-0030	*COL1A2*	NM_000089.4	Het	SNV	c.1685G>A	p.(Gly562Asp)	P	VarID:988496	AD	De novo	No
6	M	20 + 3	Lung	Osteogenesis imperfecta, progressively deforming (Sillence type 3), *COL1A2*-related	26-0090	*COL1A2*	NM_000089.4	Het	SNV	c.2819G>T	p.(Gly940Val)	P	VarID:988398	AD	De novo	No
7	M	18+	Lung	Osteogenesis imperfecta, progressively deforming (Sillence type 3), *COL1A2*-related	26-0090	*COL1A2*	NM_000089.4	Het	SNV	c.2234G>A	p.(Gly745Glu)	LP	VarID:3363155	AD	De novo	No
8	M	18 + 1	Skin	Achondrogenesis, *COL2A1*-related (formerly type 2, type Langer-Saldino)	02-0010	*COL2A1*	NM_001844.5	Het	SNV	c.3437G>A	p.(Gly1146Asp)	LP	VarID:988366	AD	De novo	No
9	M	19+	Lung	Kniest dysplasia, *COL2A1*-related	02-0060	*COL2A1*	NM_001844.5	Het	SNV	c.1266+1G>T	p.?	P	VarID:2687874	AD	De novo	No
10	M	17+	Lung	Achondrogenesis, *COL2A1*-related (formerly type 2, type Langer-Saldino)	02-0010	*COL2A1*	NM_001844.5	Het	Indel	c.3062_3079del	p.(Pro1021_Gly1026del)	LP	VarID:438682	AD	NT	No
11	F	13+	Lung	Achondrogenesis, *COL2A1*-related (formerly type 2, type Langer-Saldino)	02-0010	*COL2A1*	NM_001844.5	Het	SNV	c.3077G>A	p.(Gly1026Asp)	P	VarID:2687876	AD	De novo	No
12	M	19+	Lung	Short rib-polydactyly syndrome (SRPS), *DYNC2H1*-related	10-0010	*DYNC2H1*	NM_001080463	Het comp	SNV	c.[7129T>G] c.[6478-16G>A]	p.(Phe2377Val) r.6477_6478ins6478-14_6478-1	VUS (PM2, PM3, PP3) LP	VarID:558751 VadID:558752	AR	Ma Pa	Yes, PMID:33875766, 31965514
13	M	18+	Lung	Short rib-polydactyly syndrome (SRPS), *DYNC2H1*-related	10-0010	*DYNC2H1*	NM_001080463	Het comp	SNV Indel	c.[10163C>T] c.[2386del]	p.(Pro3388Leu) p.(Arg796Glyfs*8)	P P	VarID:439631 VarID:638016	AR	Pa Ma	No
14	F	20+	Lung	Thanatophoric dysplasia (type 1), *FGFR3*-related	01-0010	*FGFR3*	NM_000142.5	Het	SNV	c.742C>T	p.(Arg248Cys)	P	VarID:16332	AD	NT	No
15	M	13+	Lung	Thanatophoric dysplasia (type 1), *FGFR3*-related^d^	01-0010	*FGFR3*	NM_000142.5	Het	SNV	c.1118A>G	p.(Tyr373Cys)	P	VarID:16342	AD	NT	No
16	F	19+	AF	Thanatophoric dysplasia (type 1), *FGFR3*-related	01-0010	*FGFR3*	NM_000142.5	Het	SNV	c.1118A>G	p.(Tyr373Cys)	P	VarID:16342	AD	NT	No
17	F	20+	Lung	Thanatophoric dysplasia (type 1), *FGFR3*-related	01-0010	*FGFR3*	NM_000142.5	Het	SNV	c.742C>T	p.(Arg248Cys)	P	VarID:16332	AD	NT	No
18	F	35+	CVB	Achondroplasia, FGFR3-related	01-0040	*FGFR3*	NM_000142.5	Het	SNV	c.1138G>A	p.(Gly380Arg)	P	VarID:16327	AD	NT	No
19	M	19+	AF	Thanatophoric dysplasia (type 1), *FGFR3*-related	01-0010	*FGFR3*	NM_000142.5	Het	SNV	c.1108G>T	p.(Gly370Cys)	P	VarID:16359	AD	De novo	No
20	F	19+	Lung	Osteogenesis imperfecta with calcification of interosseus membranes and/or hypertrophic callus (OI type 5), *IFITM5*-related	26-0350	*IFITM5*	NM_001025295.3	Het	SNV	c.119C>T	p.(Ser40Leu)	P	VarID:183677 PMID: 26031935.	AD	De novo	Yes
21	M	34+	AF	Short-rib thoracic dysplasia^e^	10	*IFT74*	NM_001099222.3	Hom	Del	chr9:26959922_26962977del	r.-19_120del	LP	VarID:984954 PMID 33875766, 37315079	AR	Par het	Yes, PMID 33875766
22	F	20+	AF	Opsismodysplasia, *INPPL1*-related	14-0050	*INPPL1*	NM_001567.4	Hom	SNV	c.1397T>A	p.(Ile466Asn)	VUS (PM2, PP3)	VarID:2687875	AR	Par het	Yes
23	M	13+	CVB	Greenberg dysplasia, *LBR*-related	23-0050	*LBR*	NM_002296.4	Hom	SNV	c.1127C>G	p.(Pro376Arg)	VUS (PM2, PP3, PP4)	VarID:2599968	AR	Par het	Yes
24	F	29+	Lung	Focal dermal hypoplasia (Goltz Syndrome), *PORCN*-related	39-0150	*PORCN*	NM_203475.3	Het	SNV	c.887G>C	p.(Arg296Pro)	LP	VarID:2090560	XLD	De novo	No
25	M	13+	CVB	Osteogenesis imperfecta with craniosynostosis (Cole Carpenter syndrome), *SEC24D*-related	26-0370	*SEC24D*	NM_001318066.2	Het comp	SNV Indel	c.[−167C>T] c.[791_792del]	p.? p.(Ser264Tyrfs*3)	VUS (PM2, PM3) P	VarID:3363159 VarID:988313	AR	Pa Ma	Yes
26	F	14 + 4	CVB	Acrofacial dysostosis, *SF3B4*-related (Nager syndrome)	35-0100	*SF3B4*	NM_005850.4	Het	Indel	c.788dup	p.(Pro264Thrfs*222)	P	VarID:3363154	AD	NT	Yes
27	F	12+	Lung	Campomelic dysplasia (CD), *SOX9*-related	20-0010	*SOX9*	NM_000346	Het	Indel	c.1320_1321insCACCA	p.(Asp441Hisfs*31]	P	VarID:3363156	AD	De novo	No

*AD* autosomal dominant, *AF* amniotic fluid, *AR* autosomal recessive, *comp* compound, *cons* consequence, *CVB* chorion villus biopsy, *Del* deletion, *F* female, *FS* frameshift, *GA* gestational age, *het* heterozygous, *hom* homozygous, *Indel* insertion/deletion, *Inh* inheritance, *LP* likely pathogenic, *M* male, *Ma* maternal, *Mis* missense, *NS* nonsense, *NT* not tested, *P* pathogenic, *Pa* paternal, *Par het* parents heterozygous, *SNV* single nucleotide variant, *UN* unknown, *VUS* variant of uncertain significance, *XLD* X-linked dominant, *Zyg* zygosity.

^a^Nosology of genetic skeletal disorders: 2023 revision.

^b^Richards et al. PMID 25741868.

^c^Provided in this article.

^d^47, XXY.

^e^Not in the Nosology; VarID, Variation ID in ClinVar.

### Genome sequencing and variant detection

Genome sequencing (GS) was performed on all 32 fetuses, following previously described protocols [3, 4]. For the analysis, an in silico skeletal dysplasia gene panel was applied, based on the Genomics England PanelApp (https://panelapp.genomicsengland.co.uk/panels/309/) and including genes based on the latest Nosology of Genetic Skeletal Disorders [1]. This panel, which is regularly updated to incorporate the latest genetic discoveries, was the sole method for massive parallel sequencing analysis in 26 fetuses. In the remaining six fetuses (21, 28–32), with molecularly unsolved diagnoses following the initial singleton analysis, additional evaluation was performed by trio analysis, involving the fetus, mother, and father (Supplementary Table 1). Sanger sequencing was used to validate specific variants when additional confirmation was required due to technical reasons. Validation was performed according to internal quality metrics established by the Genomic Medicine Center Karolinska Rare Disease (GMCK-RD), and was omitted when data quality and coverage were sufficient (Supplementary Table S1). Supplementary Table 2 summarizes the analyses conducted prior to massive parallel sequencing (QF-PCR and array-CGH). In most cases, array-CGH was the standard initial analysis for detecting copy number variations (CNVs). However, in nine cases, GS was used as the primary method due to fetal findings suggestive of skeletal dysplasia, and CNV analysis was performed with genome sequencing data instead of a separate array-CGH analysis (Supplementary Table S2). Structural variant (SV) analysis was performed on all samples as part of the GS workflow using the FindSV pipeline (https://github.com/J35P312/FindSV), which integrates CNVnator (v0.3.2) for copy number variant detection and TIDDIT for structural rearrangement analysis. The analysis pipeline and interpretation steps were based on previously published protocols [5–7]. GS was chosen over ES as the primary method due to its broader coverage and ability to detect a wider range of variant types, including structural and non-coding variants. Our clinic transitioned from ES to GS in 2015, and GS is now routinely used as the standard diagnostic approach for suspected monogenic disorders in both prenatal and postnatal settings. QF-PCR was utilized to exclude maternal contamination in all DNA samples obtained from CVB. For patient 28, with an unsolved diagnosis, postnatal sequencing of total blood RNA extracted from PAX tubes using standard methods was performed by Clinical Genomics at SciLifeLab, to clarify the deep intronic variants in *CEP120* identified through GS. For the variants in fetuses 12 and 21, cDNA sequencing was previously reported by our group [8]. The identified genetic variants were classified according to the American College of Medical Genetics and Genomics (ACMG) guidelines [9].

### In silico protein modeling of identified genetic variants

Protein information and structures were obtained from NCBI [10], Ensembl [11], UniProt [12], PDB [13, 14] and AlphaFold2 [15, 16] databases. Protein structures with PDB codes 7YIW [17], 6SRR [18], 7URE and 7URA [19] were downloaded for the analysis. Only those genes with experimental protein structures or high-confidence AlphaFold models were considered for the structural analysis. PyMol software (The PyMOL Molecular Graphics System, Version 2.5 Schrödinger, LCC) was used to create protein images. Multiple Sequence Alignment (MSA) were performed using Clustal Omega tool [20] in UniProt webpage. *pyMSAviz* package (Shimoyama, Y., *pyMSAviz: MSA visualization python package for sequence analysis [Computer software]*, https://github.com/moshi4/pyMSAviz 2022) was used to prepare MSA figures.

## Results

### Diagnostic yield and variant classification

We identified pathogenic (P) or likely pathogenic (LP) variants in 23 out of 32 fetuses, achieving a diagnostic yield of 72%. In total, we report on 31 variants in fetuses considered molecularly solved in this cohort, including 19 pathogenic, eight likely pathogenic and four variants of uncertain significance (VUS). All four VUS were identified in fetuses diagnosed with conditions that follow an autosomal recessive (AR) inheritance pattern. In two fetuses, the VUS co-occurred with an LP or P variant in the same gene, and in two, the VUS was found in homozygosity. The ACMG criteria applied are provided in Table 1.

**Fig. 1:**
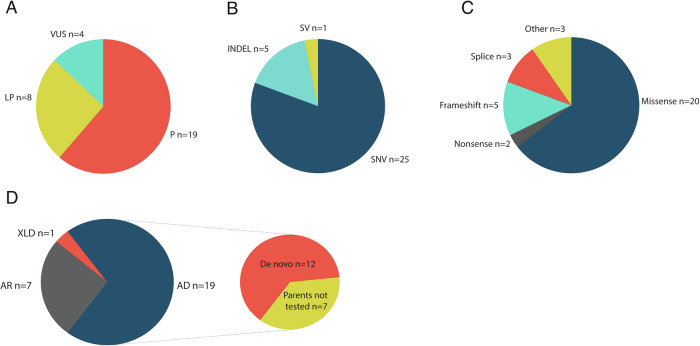
Variant classification, coding consequences, inheritance patterns and variant origins of the molecularly solved fetuses in the cohort. Each unique variant is counted once per patient if present in a homozygous state. **A** Classification and proportions of variants of uncertain significance (VUS), likely pathogenic (LP), and pathogenic (P). A total of 31 variants were detected, eight likely pathogenic, 19 pathogenic variants, and four VUS. **B** Categorization of the variants based on their type: structural variant (SV), single nucleotide variant (SNV), or insertion/deletion (INDEL). In total, 31 variants were detected; out of these, the majority were SNVs. **C** Predicted coding consequences of the variants, categorized into missense, nonsense, frameshift, splice site, and other variants. The “other” category includes the prediction of a new start codon in *SEC24D*, an intragenic deletion in *IFT74* and an in-frame indel in *COL2A1*. **D** Inheritance patterns and variant origins in the cohort. Inheritance patterns are categorized into autosomal dominant (AD), autosomal recessive (AR), and X-linked dominant (XLD), the majority being AD inheritance (19 out of 27 cases, corresponding to 70%). Among the AD cases, 12 were confirmed as de novo occurrences, and in an additional seven cases, de novo inheritance was assumed as neither of the parents exhibited the phenotype. Each fetus diagnosed with an AR condition, whether homozygous or compound heterozygous variants, is counted once. The variants identified in fetus number 28 have been excluded from the AR category, as this case remains molecularly unsolved. For the AR conditions (*n* = 7), all parents (*n* = 14) were tested and confirmed heterozygous carriers of the variants. Three fetuses carried homozygous variants, and four had compound heterozygous status. The XLD condition (fetus 24) was inherited de novo, confirmed by segregation analysis.

Genome sequencing results are summarized in Table 1, Fig. 1 and Supplementary Table 2.

### Previously unreported variants

Of the total 31 variants detected in the molecularly solved fetuses, 15 were previously reported in ClinVar database or scientific literature in PubMed (Table 1, Supplementary Table 2), and 16 were unreported.

### Clinical characteristics and radiographic diagnoses

**Fig. 2:**
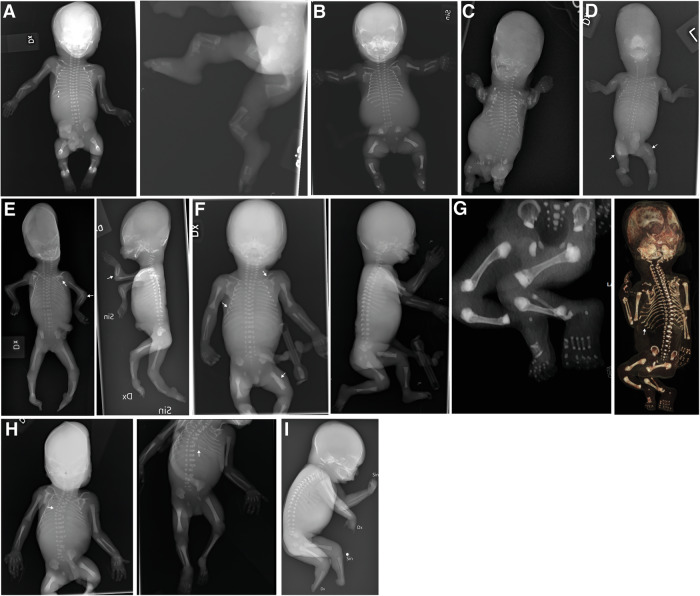
Skeletal radiographs of selected fetuses in the study with rare phenotypes and unsolved molecular diagnoses. **A** Fetus 20, 19 weeks. OI type 5, *IFITM5*-related (OMIM#610967). Note mildly reduced mineralization with several bilateral rib and left clavicle fractures (arrows), some with callus formation. Steep angulation of long tubular bones, except for humerus. **B** Fetus 22, 20+ weeks. Opsismodysplasia, *INPPL1*-related (OMIM#258480). Note severe platyspondyly, iliac hypoplasia, short tubular bones with irregular metaphyses and remarkable brachydactyly in hands and feet. **C** Fetus 23, 13 weeks. Greenberg dysplasia, *LBR*-related (OMIM#215140). Note irregular mineralization. Severely short long tubular bones with anarchic ossification. Thorax hypoplasia with gracile irregular ribs. Low mineralization of vertebral bodies. **D** Fetus 25, 14 weeks. Cole-Carpenter syndrome, *SEC24D*-related (OMIM#616294). Note low mineralization, short extremities and multiple fractures of the ribs and tubular bones (arrows). **E** Fetus 26, 16 weeks. Acrofacial dysostosis, *SF3B4*-related (Nager syndrome OMIM#154400). Note retromicrognathia, left scapular hypoplasia (arrow), elbow misalignment, bilateral radioulnar synostosis (arrows), ulnar deviation in wrists, bilateral absence of thumbs, bent tibia, right fibular hypoplasia, left fibular aplasia, and bilateral pes equinovarus. **F** Fetus 29, 18 weeks. OI, unsolved molecular diagnosis. Note decreased mineralization of skull and face. Left femoral fracture with callus (arrow). Few rib and left clavicular fractures (arrows). Markedly slender diaphyses of the long tubular bones. **G** Fetus 30, 17 weeks. Metaphyseal osteosclerosis, unsolved molecular diagnosis. Postmortem CT. Note severe sclerosis of the metaphyses in tubular bones, metaphyseal equivalents and in vertebral bodies. Slender ribs with bilateral fractures (arrow). **H** Fetus 31, 20 + 1 weeks. Spondylocostal dysostosis, unsolved molecular diagnosis. Note multiple segmentation anomalies (arrow), leftsided partial dorsal rib fusions (costae 6–7 and 8–10) (arrow), and unilateral left acetabular dysplasia. **I** Fetus 32, 20 weeks. Peromelia/ectrodactyly, unsolved molecular diagnosis. Note reduction anomalies of all four extremities. Absent feet. Left hand with shortened fingers 2–4 and partial syndactyly, right hand with shortened fingers 1 and 5, and stumps of fingers 2–4.

The most prevalent diagnostic category was the Osteogenesis Imperfecta (OI) and bone fragility group, accounting for nine cases. This group included three cases each of OI type 2 and type 3, and three fetuses with other rare forms of OI. Among these other forms is Cole-Carpenter syndrome, *SEC24D*-related (OMIM#616294), an extremely rare form presenting with osteopenia, short and bent bones with multiple fractures, and a deformed unmineralized skull (Fig. 2D). This fetus’s clinical findings were broad fontanels, low-set ears, and mild hypertelorism, identified during fetal autopsy. The other two cases were OI type 5, *IFITM5*-related, (OMIM#610967), observed in fetus 20 with severe angular deformity of the femurs, lower legs, and forearms (Fig. 2A) and a molecularly unsolved gracile bone dysplasia seen in fetus 29 (Fig. 2F), which is discussed further below.

The second most common diagnostic category is *FGFR3*-related chondrodysplasias, with six diagnosed cases: five thanatophoric dysplasia type 1 (TD1) and one achondroplasia. TD1 is the most frequently diagnosed individual condition within our cohort.

In total, 26 pregnancies were terminated by choice of the parents, while two resulted in missed abortions. One fetus (case 21) was homozygous for an intragenic deletion in *IFT74* and died shortly after birth [8]. Case 28 remains an unsolved diagnosis of orofaciodigital syndrome, described in more detail below, and case 18 was diagnosed with achondroplasia.

**Fig. 3:**
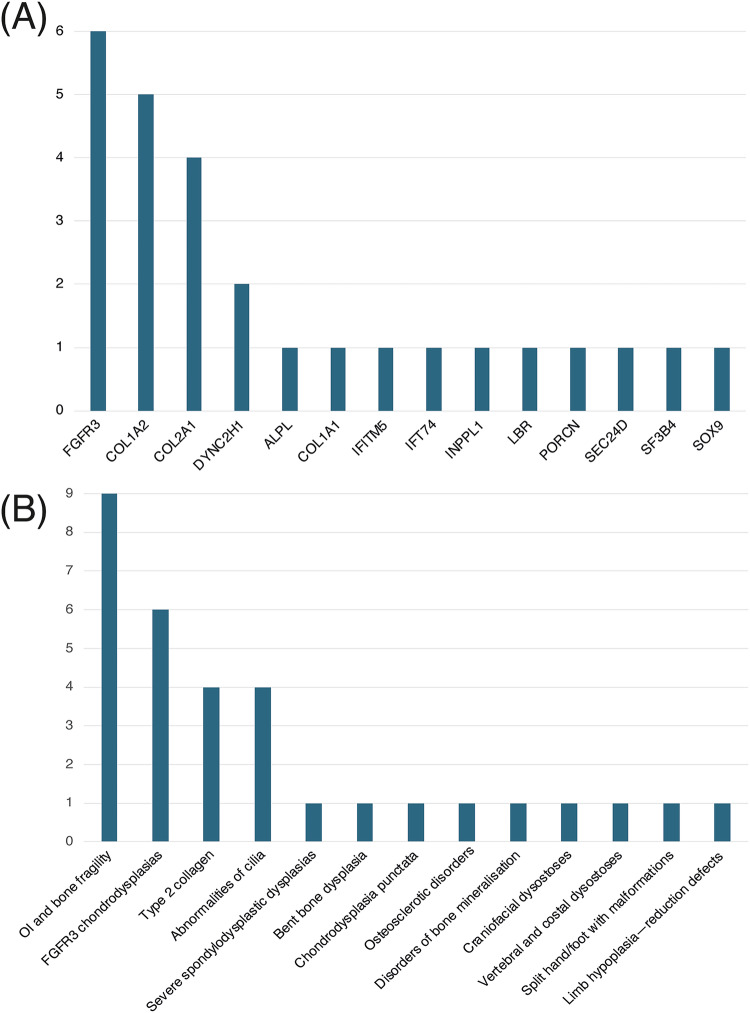
Genes and diagnostic groups. **A** The frequency of the genes in which variants were identified within our cohort, illustrating the genetic diversity of the disorders. **B** Cases are categorized into the diagnostic groups as defined in the Nosology 2023 [1]. Bars represent the number of cases per diagnostic category, illustrating the prevalence of each diagnostic group in our study.

**Fig. 4:**
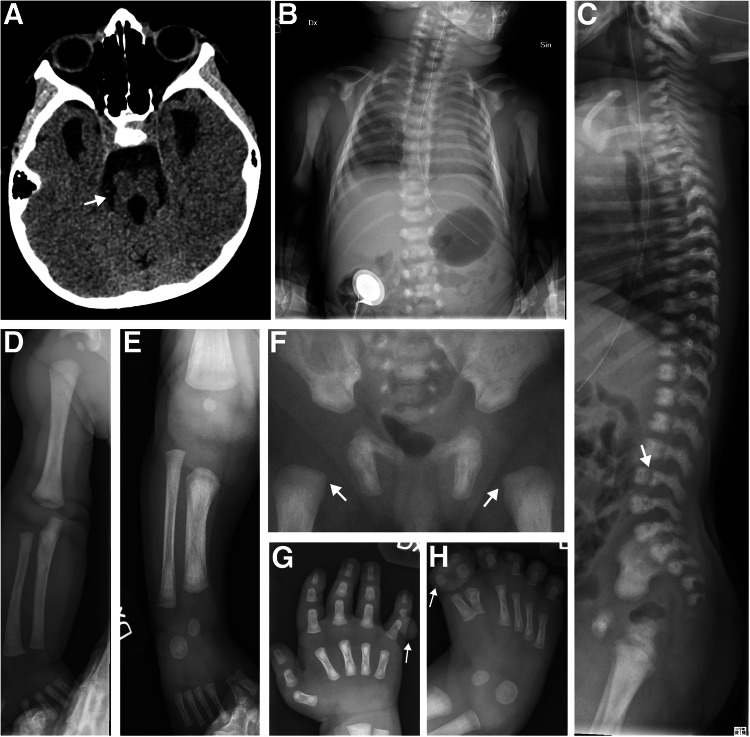
Skeletal radiographs of patient 28 with a clinical diagnosis of orofaciodigital syndrome. CT at 3 years and radiographs at 1 month of age. **A** Cerebral CT, showing molar tooth sign (arrow). **B** Chest, thoracic cage is within normal range. **C** Lateral spine, with normal height of vertebral bodies. Note sporadic coronal clefts (arrow). **D** Right upper extremity, normal. **E** Lower right extremity shows short tibia and broad diaphyses. Proximal tibial epiphysis is not ossified. **F** Pelvis, normal. Note short femoral necks (arrows). **G** Right hand with a rudimental postaxial extra finger (arrow). **H** Foot, note preaxial polydactyly (arrow).

The outcomes of all pregnancies are summarized in Table 1, Figs. 1 and 3, where we categorize them according to the Nosology 2023 [1]. Radiographs of nine fetuses are provided in Fig. 2. Radiographs of fetus numbers 12 and 21 have already been published [8, 21]. For case 28, the radiographs were obtained postnatally (Fig. 4).

### Protein structural analyses

To support potential involvement of specific genetic variants in the observed phenotypes, we conducted structural protein analyses in silico. Four selected missense variants, two VUS (in *INPPL1* and *LBR)*, and two LP (in *ALPL* and *PORCN)*, were examined (data shown in Supplementary Figs. S2 and S3). Fetus 22 with opsismodysplasia, has a homozygous *INPPL1* missense variant p.(Ile466Asn), classified as VUS. *INPPL1* encodes SHIP2, a phosphatase involved in phosphatidylinositol signaling. The affected residue is located in a conserved phosphatase domain [22] between two ß-sheets in a hydrophobic environment. Substituting isoleucine with the polar amino acid asparagine likely disrupts hydrophobic interactions, affecting protein folding and function. Fetus 23 with Greenberg dysplasia, has a homozygous *LBR* variant p.(Pro376Arg), classified as VUS. This gene encodes lamin B receptor, an inner nuclear membrane protein involved in cholesterol biosynthesis and nuclear structure. The affected residue is located in the highly conserved sterol reductase domain [23]. The substitution of a small, hydrophobic proline, with a longer, positively charged arginine disrupts interactions between a conserved loop near the catalytic site, and one of the alpha helix (Supplementary Fig. S3B). Additionally, structural analysis of LP variants found in the *ALPL* (p.Asn124Asp) and *PORCN* (p.Arg296Pro) genes (found in fetus 1 and 24, respectively) further support their contribution to the phenotypes. *ALPL* encodes the enzyme tissue-nonspecific alkaline phosphatase (TNAP), involved in bone mineralization. The p.(Asn124Asp) variant is found in compound heterozygosity with a nonsense variant in a fetus with hypophosphatasia (fetus 1). Asn124 is highly conserved across species and homologous alkaline phosphatases, and forms hydrogen bonds with Ala132 and Thr134 (Supplementary Fig. S2B, C). Substituting asparagine with aspartate disrupts these bonds, which are important for structural stabilization of a loop at the dimerization interface, likely resulting in a dysfunctional protein [17]. The functional importance of Asn124 is further supported by a previously reported missense variant at the same codon, p.(Asn124Ser), which showed significantly reduced enzyme activity (0.26 relative activity and 0.53 WT/combined activity) in in vitro studies, according to the LOVD database [24]. Fetus 24, with focal dermal hypoplasia, has a heterozygous *PORCN* variant, p.Arg296Pro, classified as LP. The encoded protein, porcupine, is a membrane-bound enzyme that regulates the Wnt signaling pathway by attaching a palmitoleoyl group from palmitoleoyl-CoA to a conserved serine on Wnt proteins, required for their interaction with Frizzled receptors [19, 25]. The highly conserved Arg296 interacts with a zinc-binding loop, and a substitution of arginine to proline would disrupt this interaction (Supplementary Fig. S3C). This is similar to the known pathogenic variant, p.Ser297Leu, which also disrupts the interaction between serine and the zinc-loop, indicating a significant impact on protein function [26].

### Trio and unsolved molecular cases

Initially, six fetuses remained molecularly unsolved after skeletal dysplasia gene panel analysis as singletons, as outlined in the flowchart (Supplementary Fig. S1). Following trio analysis of genome sequencing data from these fetuses and their parents, one additional diagnosis was resolved, identifying a likely pathogenic homozygous intragenic deletion in the *IFT74* gene (fetus 21) associated with short-rib thoracic dysplasia [8]. Diagnoses for five fetuses remain unsolved molecularly. A summary of their clinical and radiological features, along with genetic analysis results, is provided in Table 2, with radiographs shown in Figs. 2 and 4. In one unsolved case with a complex skeletal ciliopathy phenotype (fetus 28), we identified variants in the *CEP290* and *CEP120* genes, as detailed below, but they cannot fully explain the observed phenotype. The trio analysis revealed one pathogenic variant in the *CEP290* gene, NM_025114.4 c.(164_167del), p.(Thr555Serfs*3), and one VUS in *CEP120* NM_153223.4 c.(2917C>T), p.(Arg947*). Both these genes are known to be involved in ciliopathies, which could suggest an involvement in the phenotype. Searching for possible compound variants, we also identified two intronic *CEP120* variants, NM_153223.4, c.(−33+214C>T) and c.(2580+5133T>A), which were absent in the gnomAD database and situated on the alternate allele from the previously identified variant in *CEP120*, but cDNA analysis using RNA from the patient’s blood showed no splicing effect, and their impact on the phenotype remains unclear.

**Table 2:** Summary of clinical and genetic features for each of the five fetuses where a conclusive molecular diagnosis could not be achieved.

Nr	Clinical diagnosis	Sex	GA	Tissue	Clinical features	Molecular findings
28	Orofaciodigital syndrome	M	16+	AF, blood	Born at 37 weeks to healthy non-consanguineous parents. Antenatal detection of skeletal and intracranial abnormalities and polyhydramnios. Microcephaly, microretrognathia, tongue hamartoma. Small hands with broad big toes and thumbs. Postaxial polydactyly/soft tissue of the hands. Preaxial polydactyly of the feet.	*CEP290*: NM_025114.4 c.(164_167delCTCA), p.(Thr555Serfs*3) inherited from father *CEP120:* NM_153223.4 c.(2917C>T), p.(Arg947*) inherited from mother. Two intronic variants inherited from father: c.(−33+214C>T) and c.(2580+5133T>A)
29	Osteogenesis imperfecta	M	18+	CVB	Terminated at week 19. Healthy non-consanguineous parents, first pregnancy. Ultrasound at GA 18 + 2 showed a suspected left femur fracture and short long bones. Autopsy confirmed an older femur fracture and thinner diaphysis, no dysmorphic features, and no inner organ anomalies. No reports on decreased fetal movements during pregnancy.	No variant detected
30	Metaphyseal osteosclerosis	M	17+	Fetal tissue	Missed abortion at week 17. Healthy non-consanguineous parents, four previous early miscarriages.	No variant detected
31	Spondylocostal dysostosis	M	20 + 1	Fetal tissue	Terminated at 20 weeks. Healthy non-consanguineous parents. Multiple segmentation anomalies, scoliosis, left hip dislocation, and overlapping fingers on the left hand. No dysmorphic features. Abnormal external genitalia, anal atresia, absent left kidney and ureter, with right kidney showing mild hydronephrosis and abnormal ureter.	No variant detected
32	Ectrodactyly, peromelia	M	20+	Fetal tissue	Terminated at 20+ weeks. Bilateral absence of feet and left hand with shortened fingers 2–4 and partial syndactyly between digits 4 and 5. Right hand with shortened fingers 1 and 5, and stumps of fingers 2–4. Mild bilateral hydroureter.	No variant detected

*AF* amniotic fluid, *CVB* chorionic villus biopsy, *GA* gestational age, *M* male.

## Discussion

### Diagnostic yield, gene panel efficacy and variant interpretation

Genome sequencing in this cohort identified pathogenic or likely pathogenic variants in 72% of the fetuses examined (23 out of 32), demonstrating a high diagnostic yield. Additionally, when including the four VUS strongly suspected to be causative for the observed phenotype, the overall detection rate increased to 84% (27/32). To better understand how GS contributed to the diagnosis, we evaluated whether a correct clinical diagnosis could be made based on prenatal imaging, autopsy and radiological findings alone, before the genetic results were available. In most cases (*n* = 26), clinical assessment including radiology and autopsy was performed before GS. However, in six fetuses, genome sequencing results were available rapidly during the ongoing pregnancy, and therefore preceded autopsy and radiological examination. Excluding these six from the comparison between clinical and genomic diagnostic yield, we found that in 17 of the 26 fetuses (65%), a correct clinical and radiological diagnosis was established prior to genome sequencing and later confirmed genetically through the identification of a pathogenic or likely pathogenic variant in the anticipated gene. When including cases with VUS, this number increased to 18 fetuses (69%), indicating that over half of the diagnoses were correct based on radiological and autopsy findings alone. Most of these cases (*n* = 7) were classified within the nosology group 26 (osteogenesis imperfecta), three within group 1 (FGFR3), and one case each in groups 10, 20, 35 and 39. In three fetuses (number 12, 15 and 22; 12%), the initial clinical diagnosis was revised after genome sequencing due to the identification of a variant (P, LP or VUS) inconsistent with the preliminary diagnosis. Thus, when comparing only the 26 fetuses in which GS was performed after clinical assessment, including radiology and autopsy, GS increased the diagnostic yield from 69% (18/26) to 81% (21/26).

The gene panel proved to be highly effective, with a 68% detection rate for identifying LP or P variants identified in 22 out of the 32 fetuses. By applying trio analysis to the six unsolved cases, we resolved one additional case by identifying a LP homozygous intragenic deletion in the *IFT74* gene, associated with short-rib thoracic dysplasia (fetus 21), as previously published [8]. This finding also illustrates the advantage of GS over ES in this setting, as this variant would not be detected by ES due to its limited coverage of non-coding regions and lower resolution for structural variants. Four of the VUS, identified in *DYNC2H1*, *INPPL1*, *LBR* and *SEC24D* (Table 1), are considered strong potential causative variants, as they were identified in genes associated with the observed clinical conditions, supported by specific radiographic features, as well as their impact on the predicted protein structure. The VUS in fetus 25, *SEC24D* c.-167C>T, is hypothesized to introduce a new start codon, potentially extending the protein’s reading frame to include previously untranslated regions.

### Role of protein structural analysis in evaluating genetic variants

Our structural analysis of selected missense variants provides insights into the potential underlying mechanisms by visualizing and predicting how these changes affect protein structure. By focusing on genes with experimental protein structures or high-confidence AlphaFold models, the predictions are based on reliable structural data. Structural analysis of the variants in INPPL1 and LBR, both classified as VUS, indicates that they are located within critical functional domains, supporting their pathogenicity. For fetus 22 with opsismodysplasia, the p.Ile466Asn in SHIP2, the protein encoded by *INPPL1*, is located in the phosphatase domain [22]. Although this variant is classified as a VUS, our structural analysis supports its pathogenicity as the isoleucine to asparagine substitution is predicted to disrupt essential hydrophobic interactions within a conserved domain (Supplementary Fig. S3A). Similarly, in fetus 23 with Greenberg dysplasia, the p.(Pro376Arg) variant in LBR, also classified as a VUS, interferes with conserved interactions within the sterol reductase domain, potentially affecting the enzymatic activity (Supplementary Fig. S3B) [23].

### Clinical outcome

Approximately 200 genetic skeletal disorders can present during the fetal period, with concerns typically arising when short extremities are detected via ultrasound examination. In our cohort, TD1 and OI are the most prevalent diagnoses, even though cases with a clear and recognizable clinical phenotype were analyzed using targeted sequencing and were excluded from this study, as described in methods. Generally, prenatal ultrasound diagnosis of TD is uncomplicated in the late second trimester. However, early manifestations of TD can be variable and challenging to diagnose based on initial ultrasound findings, leading clinicians to request a broader gene panel. Similarly, an achondroplastic fetus (fetus 18) was included in this cohort. Our study shows a predominance of autosomal dominant inheritance patterns, with 61% of the cases involving de novo variants (Fig. 1D). The detection of AR conditions and carrier status in parents has significant implications for genetic counseling and decision-making in future pregnancies, considering there is a recurrence risk for the family. Notably, in three cases, the molecular diagnosis resulted in parents choosing to undergo preimplantation genetic testing (PGT) for their future pregnancies.

### Unsolved cases

Despite the advanced genetic analyses and our best efforts with a reanalysis of the trio GS data, five cases remain unsolved and continue to present diagnostic challenges. Among these five cases without a molecular diagnosis, one was diagnosed with osteogenesis imperfecta (Nosology group 26), one with spondylocostal dysostosis (Nosology group 36), one displayed severe metaphyseal osteosclerosis (Nosology group 25), and one within limb hypoplasia reduction defects group (Nosology group 38). For the unsolved case 28, diagnosed with orofaciodigital syndrome (Nosology group 10), two genetic variants have been identified, but they do not establish a conclusive molecular diagnosis. These include a pathogenic frameshift variant in the *CEP290* gene inherited from the father and a nonsense variant in the last exon of *CEP120* inherited from the mother. The variant in *CEP120* (p.Arg947*) is reported in ClinVar as pathogenic (SCV003247549.2). It is predicted to disrupt the last 14 amino acids of the protein, and not anticipated to cause nonsense mediated decay. A variant in the same region (p.Ile975Ser) was found in a homozygous fetus with a complex ciliopathy phenotype [27]. This variant has been shown to reduce the binding affinity to the C2 calcium-dependent domain containing 3 protein (C2CD3), impacting the recruitment of this protein to centrioles and impairing cilia formation [27, 28]. The *CEP120* gene has been linked to a broad range of clinical phenotypes from mild Joubert syndrome to severe conditions overlapping with multiple ciliopathies, including Meckel-Gruber syndrome, Jeune asphyxiating thoracic dystrophy, and orofaciodigital syndromes [27]. Based on the phenotypic findings in our patient, we think that *CEP120* is the most likely gene responsible for the observed phenotype in this patient. However, the absence of a compound variant, and uncertainty regarding intronic *CEP120* variants’ impact on splicing suggests that this variant alone does not fully explain the clinical presentation. We cannot exclude the possibility of an oligogenic mechanism, where both variants in *CEP120* and *CEP290* may impact the clinical presentation. Similar oligogenic contributions have been suggested in other ciliopathies, including cases with *CEP290* [29, 30].

The absence of identified genetic variants in the fetus diagnosed with spondylocostal dysostosis and presenting with multiple segmentation anomalies, scoliosis, anal atresia and absence of one kidney and ureter, is possibly due to its complex phenotype which overlaps with the VACTERL association, currently referred to as “recurrent constellation of embryonic malformations” [31]. This suggests a multifactorial etiology that may include a combination of genetic and environmental factors, rather than a single genetic basis.

Fetus number 29 displayed slender tubular bones and reduced mineralization, consistent with findings in the osteogenesis imperfecta group. No decreased fetal movements were reported during pregnancy, excluding the possibility of a slender bone phenotype associated with a fetal hypokinesia sequence.

To our knowledge, the phenotype of fetus 30 is similar to that of an ultra-rare condition metaphyseal osteosclerosis, which has been reported only once in the literature [32] and not included in the Nosology.

Our genome sequencing approach enables the detection of a broader spectrum of genetic variations, including non-coding variants that exome sequencing cannot identify. As our knowledge of genetic conditions grows, there are still challenges in understanding the genetic basis of certain conditions. The absence of identifiable variants in the five fetuses with unsolved diagnoses suggests that the cause may lie beyond detection limits for our current genetic analysis methods, potentially involving deep intronic regions, yet unidentified genes, epigenetic factors, multifactorial causes, or non-genetic factors. Diagnostic accuracy depends on detailed phenotypic data, with radiographs providing crucial information and fetal autopsy adding significant value in confirming diagnoses and correlating clinical and genetic findings, especially in cases of early presenting fetal skeletal disorders. RNA sequencing (RNAseq) is another approach for improving diagnostic accuracy. In some cases, RNAseq could add valuable information into gene expression profiles and uncover pathogenic mechanisms from genome sequencing data alone. Challenges such as RNA quality and preservation in fetal tissues, as well as the complexity of interpreting gene expression patterns specific to developmental stages, may limit its applicability. Tissue samples were available for some fetuses in this cohort, and future studies could explore integrating RNA-seq into the diagnostic workflow to address the unsolved cases and deepen our understanding of the underlying mechanisms. Therefore, continuous advancements in genetic testing technologies are essential, and we recommend reanalysis of the genome data within 1–2 years for these families.

## Supplementary information

Supplemental material

## Data Availability

In accordance with European law, as outlined in the General Data Protection Regulation (GDPR) (https://eur-lex.europa.eu/eli/reg/2016/679/oj), the sharing of entire genome sequencing datasets from European patients is prohibited. Sharing raw or modified genome sequencing datasets is not included in our ethical permit or the informed consent signed by the patients. However, anonymized limited clinical data is available by contacting the corresponding authors and will be provided within two weeks from the request. All variants identified in this study have been submitted to the ClinVar database (Supplementary Table 2). We are prepared to share small subsets of variants of interest upon a reasonable request to the corresponding authors.
