# Supplementary material for: Genome sequencing in a cohort of 32 fetuses with genetic skeletal disorders
Source: Eur J Hum Genet. 2025 Jun 11;33(11):1474–83. doi: 10.1038/s41431-025-01886-x (PMC12583725; doi:10.1038/s41431-025-01886-x)
Supplement: Supplementary file 1 — Supplemental material [file 41431_2025_1886_MOESM1_ESM.doc]

Supplemental Material

**Supplemental Table S1.** Sequencing methods and type of analysis (singleton or trio) used in this study.

| **Table S1. Sequencing methods used in this study** | | | | | |
| --- | --- | --- | --- | --- | --- |
| **Nr** | **Method** | **Analysis** | **Platform** | **Library Prep** | **Validation** |
| 1 | GS | Singleton | NSQ | NxSeq Kit | NR |
| 2 | GS | Singleton | HSX | TruSeq Kit | Sanger |
| 3 | GS | Singleton | HSX | TruSeq Kit | Sanger |
| 4 | GS | Singleton | HSX | TruSeq Kit | Sanger |
| 5 | GS | Singleton | HSX | TruSeq Kit | Sanger |
| 6 | GS | Singleton | HSX | TruSeq Kit | Sanger |
| 7 | GS | Singleton | NSQ | TruSeq Kit | Sanger |
| 8 | GS | Singleton | HSX | TruSeq Kit | Sanger |
| 9 | GS | Singleton | NSQ | NxSeq Kit | Sanger |
| 10 | GS | Singleton | NSQ | TruSeq Kit | Sanger |
| 11 | GS | Singleton | NSQ | TruSeq Kit | Sanger |
| 12 | GS | Singleton | HSX | TruSeq Kit | Sanger |
| 13 | GS | Singleton | HSX | TruSeq Kit | Sanger |
| 14 | GS | Singleton | NSQ | TruSeq Kit | NR |
| 15 | GS | Singleton | NSQ | TruSeq Kit | NR |
| 16 | GS | Singleton | NSQ | TruSeq Kit | NR |
| 17 | GS | Singleton | NSQ | TruSeq Kit | NR |
| 18 | GS | Singleton | NSQ | TruSeq Kit | Sanger |
| 19 | GS | Singleton | NSQ | TruSeq Kit | Sanger |
| 20 | GS | Singleton | HSX | TruSeq Kit | Sanger |
| 21 | GS | Singleton+Trio | NSQ | TruSeq Kit | Sanger |
| 22 | GS | Singleton | HSX | TruSeq Kit | Sanger |
| 23 | GS | Singleton | NSQ | TruSeq Kit | Sanger |
| 24 | GS | Singleton | HSX | TruSeq Kit | Sanger |
| 25 | GS | Singleton | HSX | TruSeq Kit | Sanger |
| 26 | GS | Singleton | NSQ | TruSeq Kit | Sanger |
| 27 | GS | Singleton | NSQ | TruSeq Kit | Sanger |
| 28 | GS | Singleton+Trio | HSX | TruSeq Kit | Sanger |
| 29 | GS | Singleton+Trio | HSX | TruSeq Kit | NA |
| 30 | GS | Singleton+Trio | HSX | TruSeq Kit | NA |
| 31 | GS | Singleton+Trio | HSX | TruSeq Kit | NA |
| 32 | GS | Singleton+Trio | HSX | TruSeq Kit | NA |
| *Skeletal dysplasia gene list corresponding to Genomics England PanelApp; GS, genome sequencing; HSX, HiSeq X; NA, not applicable; NR, not required; NSQ, NovaSeq 6000; NxSeq Kit, NxSeq AmpFREE Low DNA Library Kit (Lucigen); TruSeq Kit, TruSeq DNA PCR-free | | | | | |

**Supplemental Table S2.** Genomic coordinates and pre-sequencing analyses. The genetic variants identified in the study, alongside their genomic coordinates according to the Hg38 reference, accession numbers, and variant references. It also specifies whether QF-PCR and array-CGH were performed prior to sequencing. Variants previously unreported in ClinVar or PubMed are labeled as "not reported (NR)".

| **Nr** | **Gene** | **Chr** | **Start (Hg38)** | **End (Hg38)** | **cDNA** | **Accession nr** | **Variant ref** | **QF-PCR** | **Array-CGH** |
| --- | --- | --- | --- | --- | --- | --- | --- | --- | --- |
| 1 | *ALPL* | 1 | 21563182 | 21563182 | c.370A>G | SCV005375313.1 | VarID:1721046 | Yes | No |
|  | *ALPL* | 1 | 21563271 | 21563271 | c.459G>A | SCV005375314.1 | VarID:1387764 |  |  |
| 2 | *COL1A1* | 17 | 50189510 | 50189510 | c.2696G>A | SCV001449829.1 | NR | Yes | Yes |
| 3 | *COL1A2* | 7 | 94427646 | 94427646 | c.3287G>C | SCV001449846.1 | NR | No | No |
| 4 | *COL1A2* | 7 | 94420445 | 94420445 | c.2187+1G>C | SCV005375318.1 | NR | Yes | Yes |
| 5 | *COL1A2* | 7 | 94414241 | 94414241 | c.1685G>A | SCV001450205.1 | NR | Yes | Yes |
| 6 | *COL1A2* | 7 | 94425647 | 94425647 | c.2819G>T | SCV001449951.1 | NR | Yes | No |
| 7 | *COL1A2* | 7 | 94420587 | 94420587 | c.2234G>A | SCV005375315.1 | NR | Yes | Yes |
| 8 | *COL2A1* | 12 | 47976566 | 47976566 | c.3437G>A | SCV001449842.1 | NR | No | No |
| 9 | *COL2A1* | 12 | 47987268 | 47987268 | c.1266+1G>T | SCV004232643.1 | NR | Yes | Yes |
| 10 | *COL2A1* | 12 | 47978055 | 47978072 | c.3062_3079del | SCV005375312.1 | VarID:438682 | Yes | Yes |
| 11 | *COL2A1* | 12 | 47978044 | 47978044 | c.3077G>A | SCV004232646.1 | VarID:988398 | Yes | No |
| 12 | *DYNC2H1* | 11 | 103187575 | 103187575 | c.7129T>G | SCV000788374.1 | NR, PMID 31965514 | Yes | Yes |
|  | *DYNC2H1* | 11 | 103184880 | 103184880 | c.6478-16G>A | SCV000788375.1 | NR |  |  |
| 13 | *DYNC2H1* | 11 | 103253384 | 103253384 | c.10163C>T | SCV000924634.1 | VarID: 439631 | Yes | Yes |
|  | *DYNC2H1* | 11 | 103135759 | 103135759 | c.2386del | SCV000924635.1 | NR |  |  |
| 14 | *FGFR3* | 4 | 1801837 | 1801837 | c.742C>T | SCV001450204.1 | VarID:16332 | Yes | No |
| 15 | *FGFR3* | 4 | 1804372 | 1804372 | c.1118A>G | SCV001449880.1 | VarID:16342 | Yes^1^ | Yes^2^ |
| 16 | *FGFR3* | 4 | 1804372 | 1804372 | c.1118A>G | SCV001449880.1 | VarID:16342 | Yes | No |
| 17 | *FGFR3* | 4 | 1801837 | 1801837 | c.742C>T | SCV001450204.1 | VarID:16332 | Yes | Yes |
| 18 | *FGFR3* | 4 | 1804392 | 1804392 | c.1138G>A | SCV001450245.1 | VarID:16327 | Yes | Yes |
| 19 | *FGFR3* | 4 | 1804362 | 1804362 | c.1108G>T | SCV001449937.1 | VarID:16359 | Yes | Yes |
| 20 | *IFITM5* | 11 | 299372 | 299372 | c.119C>T | SCV005375320.1 | VarID:183677, PMID: 26031935. | No | No |
| 21 | *IFT74* | 9 | 26959924 | 26962971 | chr9:26959922_26962977del | SCV001438035.2 | PMID 33875766, 37315079 | Yes | No |
| 22 | *INPPL1* | 11 | 72231089 | 72231089 | c.1397T>A | SCV004232645.1 | NR | Yes | Yes |
| 23 | *LBR* | 1 | 225411398 | 225411398 | c.1127C>G | SCV004232644.1 | VarID:2599968 | Yes | Yes^2^ |
| 24 | *PORCN* | X | 48514566 | 48514566 | c.854G>C | SCV005375324.1 | VarID:2090560 | No | Yes |
| 25 | *SEC24D* | 4 | 118836066 | 118836066 | c.-167C>T | SCV005375319.1 | NR | Yes | Yes |
|  | *SEC24D* | 4 | 118815040 | 118815041 | c.791_792del | SCV001449669 | NR |  |  |
| 26 | *SF3B4* | 1 | 149925961 | 149925961 | c.788dup | SCV005375311.1 | NR | Yes | Yes |
| 27 | *SOX9* | 17 | 72124178 | 72124182 | c.1320_1321insCACCA | SCV005375316.1 | NR | Yes | Yes |
| 28 | *CEP290* | 12 | 88140969 | 88140966 | c.164_167del | SCV005375323.1 | VarID: 217624 | Yes | Yes |
|  | *CEP120* | 5 | 123346563 | 123346563 | c.2917C>T | SCV005375321.1 | NR |  |  |
| 29 | UN | UN | UN | UN | UN | NA | NA | Yes | Yes |
| 30 | UN | UN | UN | UN | UN | NA | NA | No | Yes |
| 31 | UN | UN | UN | UN | UN | NA | NA | Yes | Yes |
| 32 | UN | UN | UN | UN | UN | NA | NA | No | Yes |
| ^1^ QF-PCR indicating Klinefelter, chromosome analysis showing 46,XXY; ^2^analysis on WGS data using the bioinformatic tool vcf2cytosure (1); NR, previously unreported at the time of initial testing; UN, unknown; Var ID, Variation ID in ClinVar. | | | | | | | | | |

**Supplemental Figure S1**: Flowchart of the study.


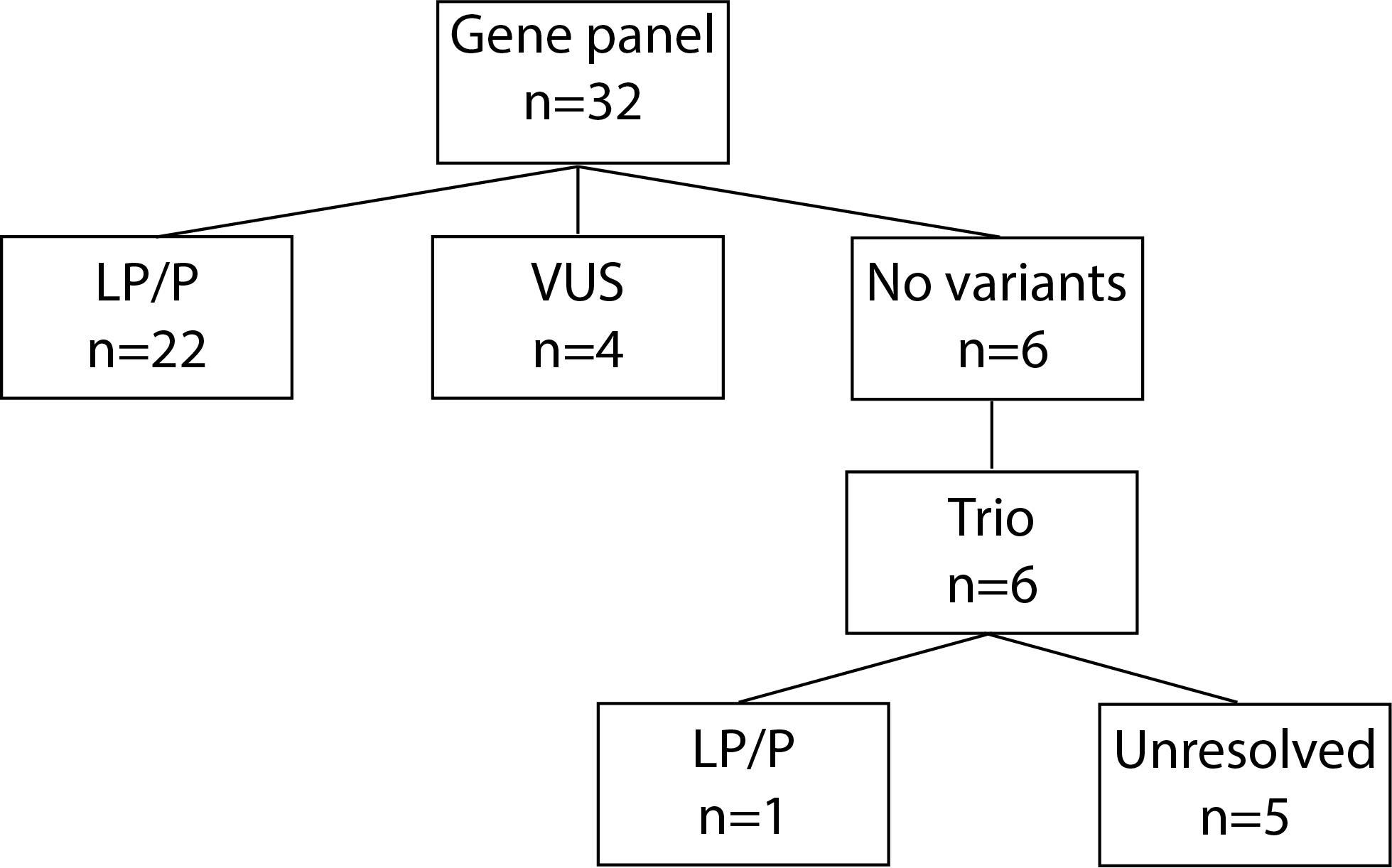


**Supplemental Figure S1.** Flowchart of the study. This chart outlines the diagnostic process and outcomes for the 32 fetuses that underwent genome sequencing. Initially, all cases were analyzed using a gene panel, resulting in 22 solved diagnoses and 4 cases with VUS considered strong. The remaining six cases with no molecular diagnosis underwent further trio analysis, which resolved one additional case. GS, genome sequencing; LP, likely pathogenic; n, number of fetuses; P, pathogenic; VUS, variant of uncertain significance.

**Supplemental Figure S2**. Predicted structural impact of TNAP variants


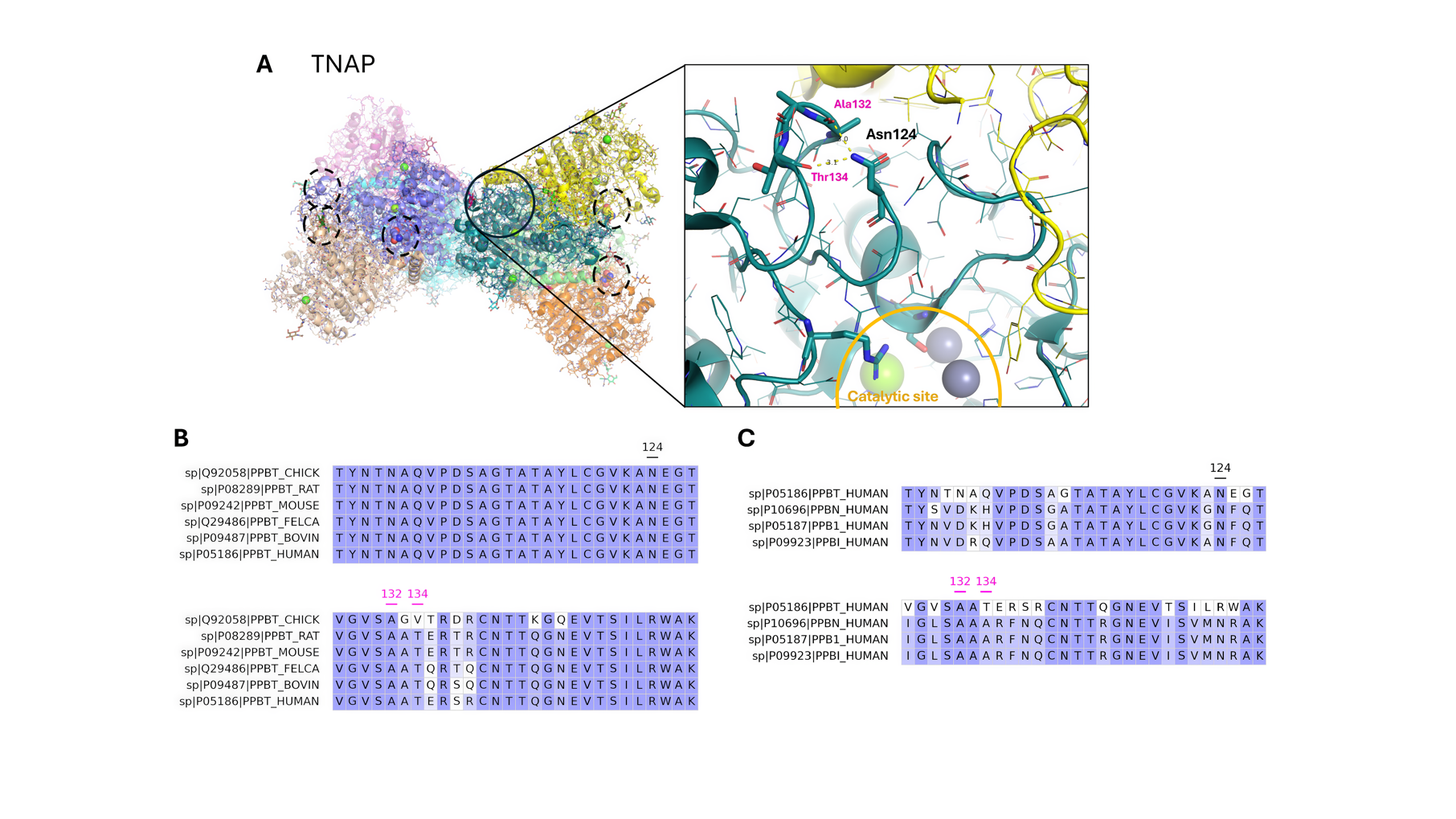


**Figure S2.** Predicted structural impact of TNAP protein variants, encoded by the *ALPL* gene. **A)** The variant at residue Asn124 is mapped onto the octameric structure of TNAP (PDB code 7YIW). Each monomer is shown in a different color, with circles indicating Asn124 on each monomer. The zoomed-in view shows Asn124 (black) and interacting residues Ala132 and Thr134 (magenta). The catalytic site is marked by an orange circle. The TNAP protein, encoded by *ALPL*, functions as a dimer and an octamer (2). The residue Asn124 is located at a dimeric interface, stabilizing a protein loop that interacts with another monomer of the protein. Substituting asparagine with aspartate is predicted to disrupt hydrogen bonds with Ala132 and Thr134, which are important for structural stabilization of a loop at the dimerization interface, likely resulting in a dysfunctional protein. The protein segment where Asn124 is located is in close contact to the catalytic site, and any destabilization could also affect the enzymatic activity. It was previously described that mutations at the dimeric interface could affect TNAP enzymatic activity and protein expression levels (2). The hypothezized destabilization caused by p.Asn124Asp is likely to occur in each dimer pair (black circles), potentially amplifying its negative effect on protein function. **B)** A portion of the sequence alignment shows residue conservation across different species and **C)** across paralogous alkaline phosphatases. Asn124 is highlighted in black in the sequence, while Ala132 and Thr134 are highlighted in magenta.

**Supplemental Figure S3.** Predicted structural impact of SHIP2, LBR and PORCN variants


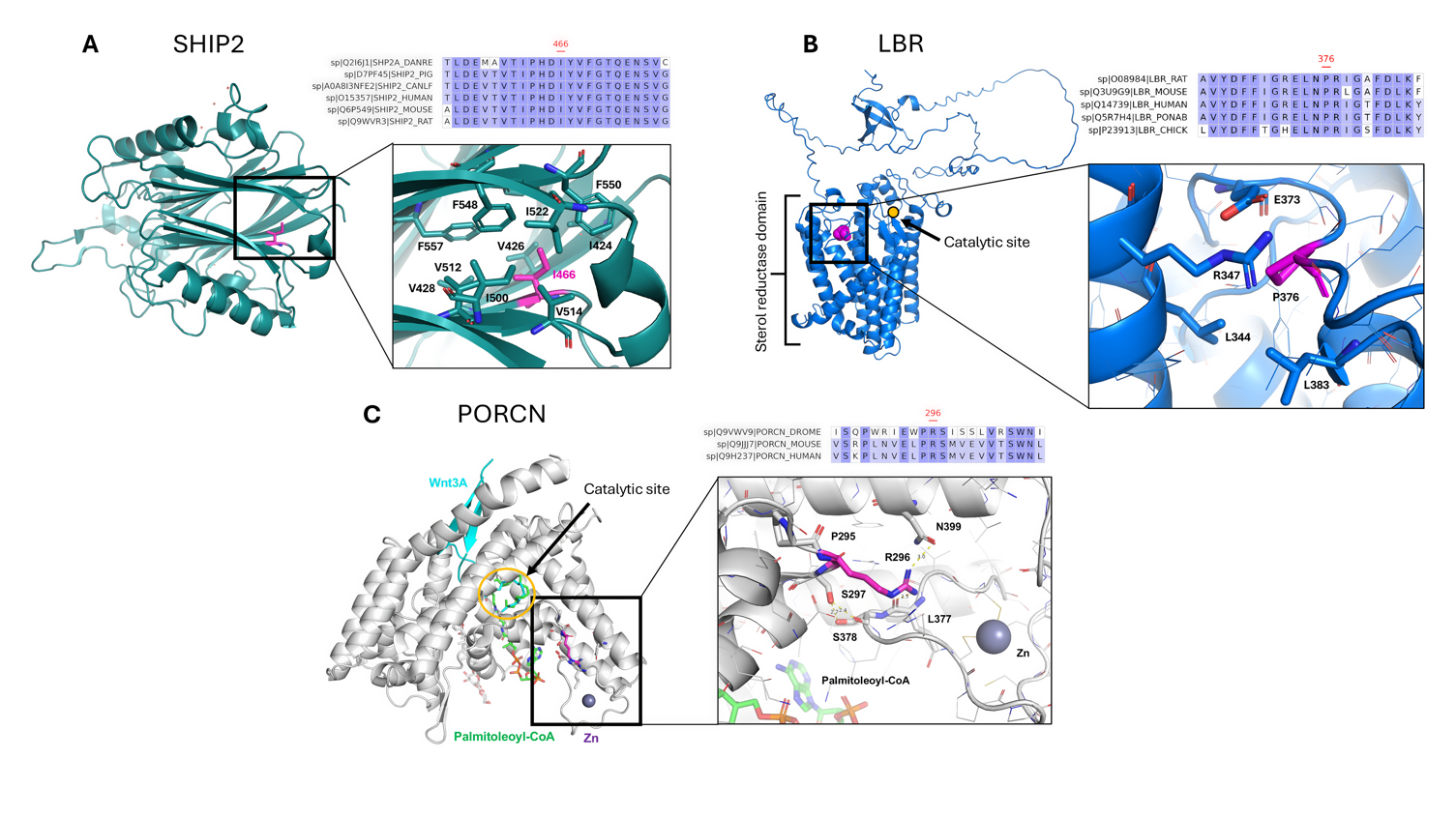


**Figure S3** Predicted structural impact of SHIP2 (encoded by *INPPL1*), LBR and PORCN variants. **A)** Experimental structure of SHIP2 (PDB code 6SRR) and a zoomed-in view showing residue interactions. A portion of the sequence alignment across different species is also shown. Ile466Asn in SHIP2 is located in the phosphatase domain. Substitution of isoleucine with asparagine is likely to disrupt essential hydrophobic interactions within this conserved domain, which in turn could affect interactions with other functional domains of the protein (3). **B)** AlphaFold model of LBR (Uniprot ID Q14739) with a zoomed-in view of residue interactions. A portion of the sequence alignment in different species is shown. **C)** Experimental structure of PORCN (PDB code 7URE) with a zoom-in of the residue interactions. A portion of the sequence alignment across different species is shown, with the location of the patient's variant highlighted in magenta in the structure and in red in the sequence. Wnt3A partial structure bound to PORCN is shown in cyan. The palmitoyl-coA compound (in green) from PDB 7URA is superposed to the structure to show the catalytic site position with respect to Arg296. The p.Arg296Pro variant is predicted to disrupt interactions that stabilize a zinc-binding loop close to the palmitoleoyl-CoA binding site. A substitution in the neighboring amino acid, Ser297Leu, has been reported as pathogenic (4). These substitutions may have similar effects on the zinc-loop interactions affecting the catalyzing function of the protein.

**References**

1. Lindstrand A, Eisfeldt J, Pettersson M, Carvalho CMB, Kvarnung M, Grigelioniene G, et al. From cytogenetics to cytogenomics: whole-genome sequencing as a first-line test comprehensively captures the diverse spectrum of disease-causing genetic variation underlying intellectual disability. Genome Med. 2019;11(1):68.

2. Yu Y, Rong K, Yao D, Zhang Q, Cao X, Rao B, et al. The structural pathology for hypophosphatasia caused by malfunctional tissue non-specific alkaline phosphatase. Nat Commun. 2023;14(1):4048.

3. Le Coq J, Camacho-Artacho M, Velazquez JV, Santiveri CM, Gallego LH, Campos-Olivas R, et al. Structural basis for interdomain communication in SHIP2 providing high phosphatase activity. Elife. 2017;6.

4. Maas SM, Lombardi MP, van Essen AJ, Wakeling EL, Castle B, Temple IK, et al. Phenotype and genotype in 17 patients with Goltz-Gorlin syndrome. J Med Genet. 2009;46(10):716-20.
